# Supplementary material for: Direct observation of deformation and resistance to damage accumulation during shock loading of stabilized nanocrystalline Cu-Ta alloys
Source: Nat Commun. 2024 Oct 23;15:9135. doi: 10.1038/s41467-024-53142-3 (PMC11499930; doi:10.1038/s41467-024-53142-3)
Supplement: Supplementary file 1 — Supplementary Information [file 41467_2024_53142_MOESM1_ESM.pdf]

"Supplementary Information for Direct observation of deformation and resistance to damage accumulation during shock loading of stabilized nanocrystalline Cu-Ta alloys"

B.C. Hornbuckle[a], R. Koju[b], G. Kennedy[c], P. Jannotti[a], N. Lorenzo[a], J. Lloyd[a], A. Giri[a], K. Solanki[d], N.N. Thadhani[c], Y. Mishin[b], and K.A. Darling[a]

[a] Army Research Directorate, DEVCOM Army Research Laboratory, APG, MD, 21005, USA
[b] Department of Physics and Astronomy, George Mason University, MSN 3F3, Fairfax, VA 22030, USA
[c] School of Materials Science and Engineering, Georgia Institute of Technology, 771 Ferst Dr. NW, Atlanta, GA 30332, USA
[d] School for the Engineering of Matter, Transport, and Energy, Arizona State University, Tempe, AZ, 85287, USA

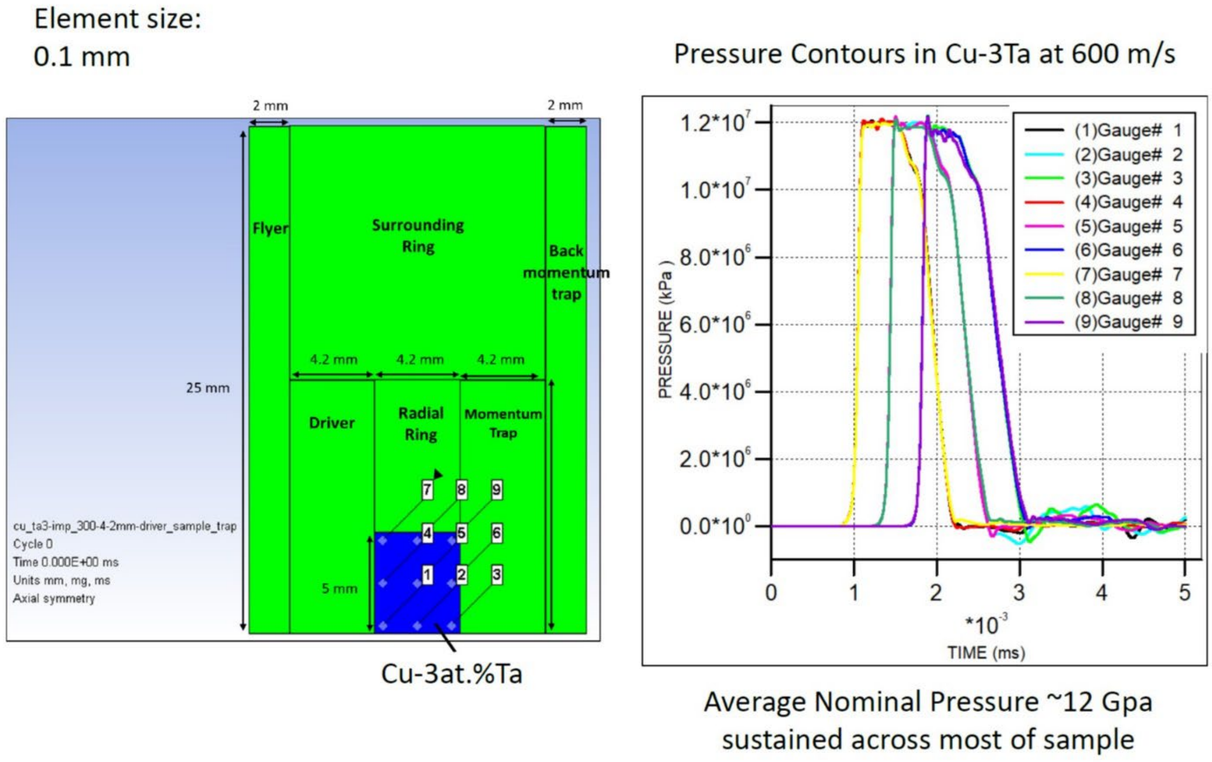

Supplementary Fig. 1: Pressure profiles generated at different locations in the target impacted at 12 GPa. The shock pressure is much higher than the HEL (2 GPa), where one would expect plasticity. This plastic deformation behavior is unlike that of ceramics, which remain in the elastic region until catastrophic failure. Additionally, PDV measurements were impossible since the fixture was designed for soft recovery, and the samples were embedded in radial and back momentum traps. However, the pulse

duration obtained from simulations is ~500 ns. Source data are provided as a Source Data file.

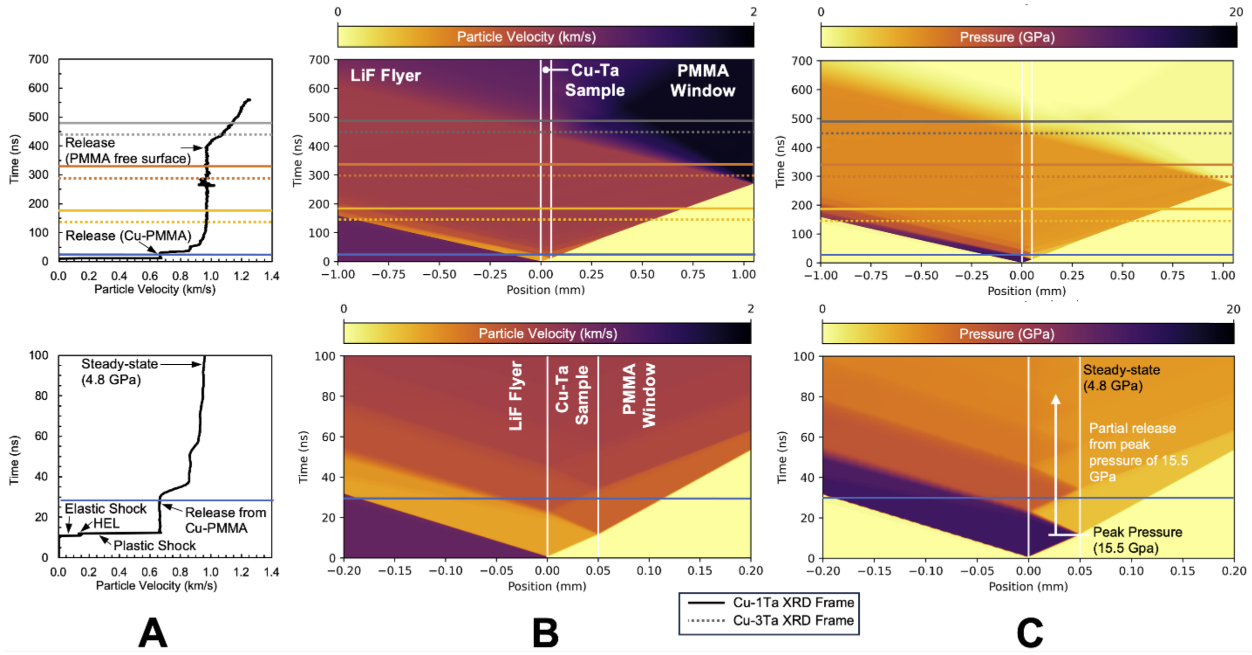

Supplementary Fig. 2: Particle velocity and pressure-time profiles for APS experiments indicative of plastic deformation (HEL ~ 2.5 GPa) overlaid with specific time stamps corresponding to the times displayed in Fig. 5. (A) Particle velocity time-history from Photonic Doppler Velocimetry shown out to approximately 600 ns and zoomed-in portion of the first 100 ns, two-dimensional model of the (B) particle velocity, and (C) pressure of the LiF flyer, Cu-Ta sample, and PMMA optical window over the full shock-release response including a zoomed-in portion of the first 100 ns after impact. According to experimental and simulated data, the Cu-Ta sample is shocked to 15.5 GPa before being partially released to 4.8 GPa. The sample becomes fully unloaded by approximately 500 ns. Source data are provided as a Source Data file.

In Figure S2, finite element simulations were used to extract the pressure and velocity-time history of the sample relative to x-ray exposure times for the experimental impact conditions. Simulations replicated the experimental configuration, wherein a 10mm thick LiF flyer traveling 1.3 km/s impacted a 50 μm thick Cu-xTa sample backed by a 1mm thick PMMA window. Since the simulations were used primarily to characterize the transient nature and order-of-magnitude estimates of velocities and pressures, existing Steinberg-Guinan equations of state were utilized for LiF, Cu, and PMMA. Figure S2C

shows that the Cu-xTa sample experiences a variable pressure-time history as the initial shock wave of 15.5 GPa from the LiF flyer transits the sample and reverberates with the PMMA window at a period of approximately 22 ns, reaching a steady pressure throughout the sample of 4.7 GPa. This pressure is maintained until the release wave from the back of the PMMA window arrives at the Cu-xTa sample approximately 450ns after the initial impact.

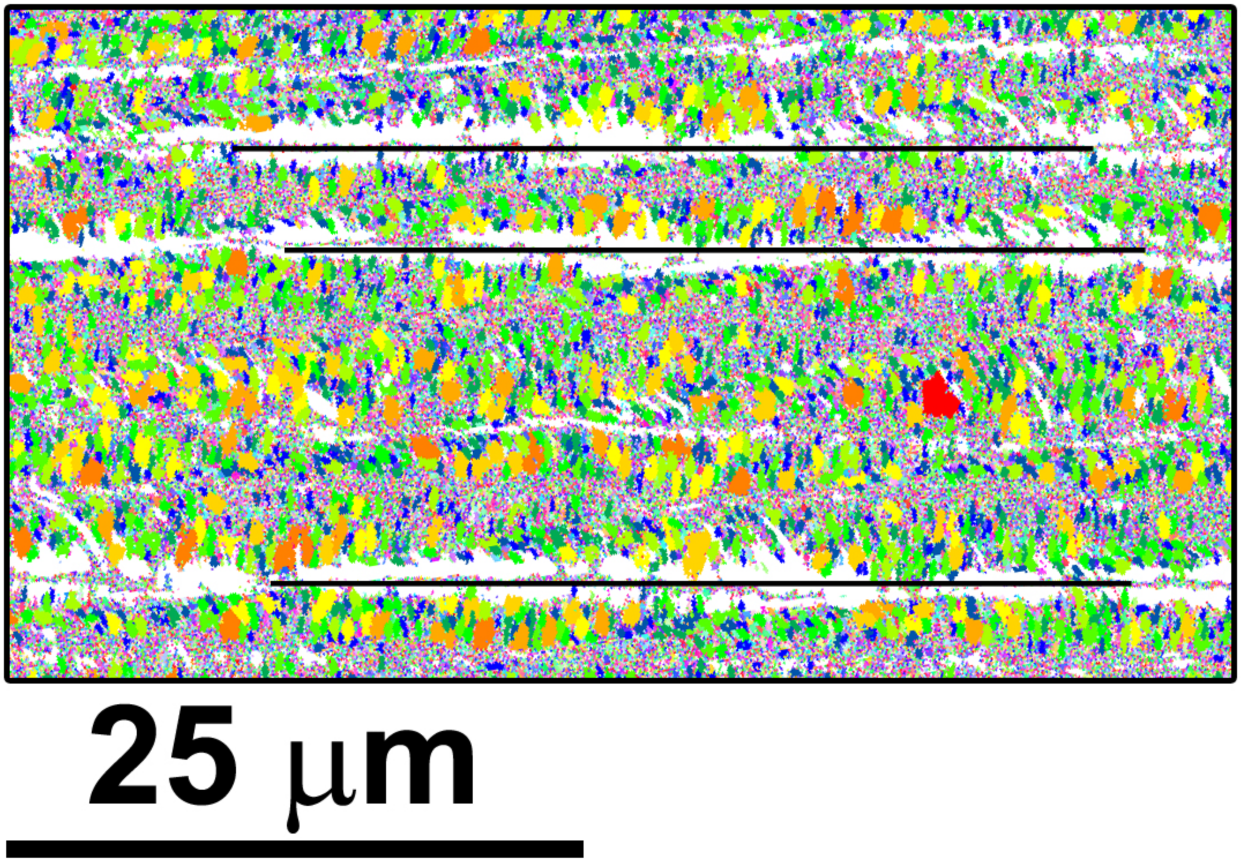

Supplementary Fig. 3: Orientation map obtained by EBSD of ECAE processed Cu sample showing significant grain growth near scratches created during the final steps of the sample polishing. Black lines mark the locations of scratches. The average increase in grain size is from ~ 150 nm to ~ 750 nm (500% increase).

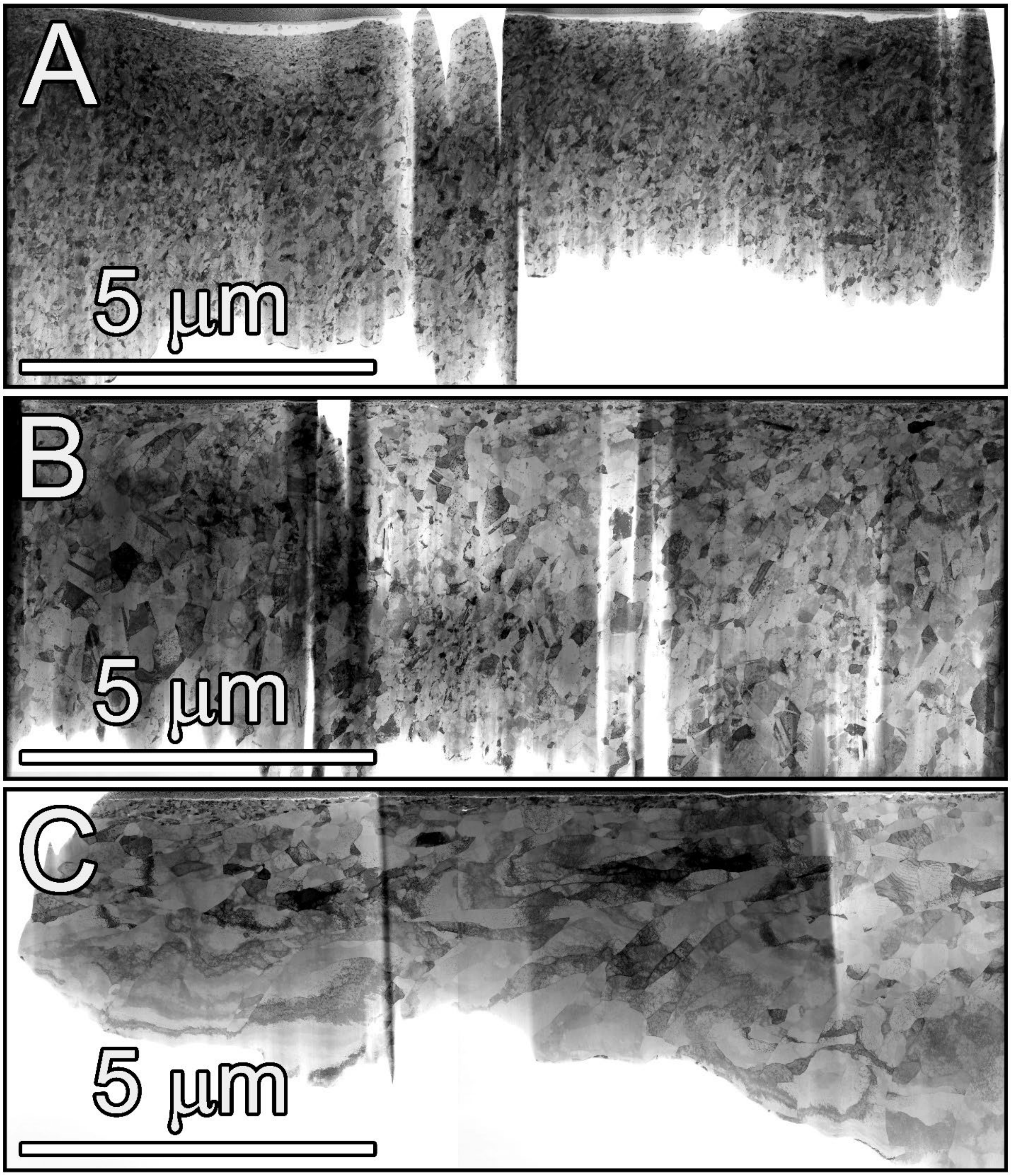

Supplementary Fig. 4: Low magnification (ultra-high-resolution) STEM bright-field images of (A) Cu-3Ta, (B) Cu-1Ta, and (C) Cu after the first shock loading. Each image comprises at least 50 square microns of electron-transparent area.

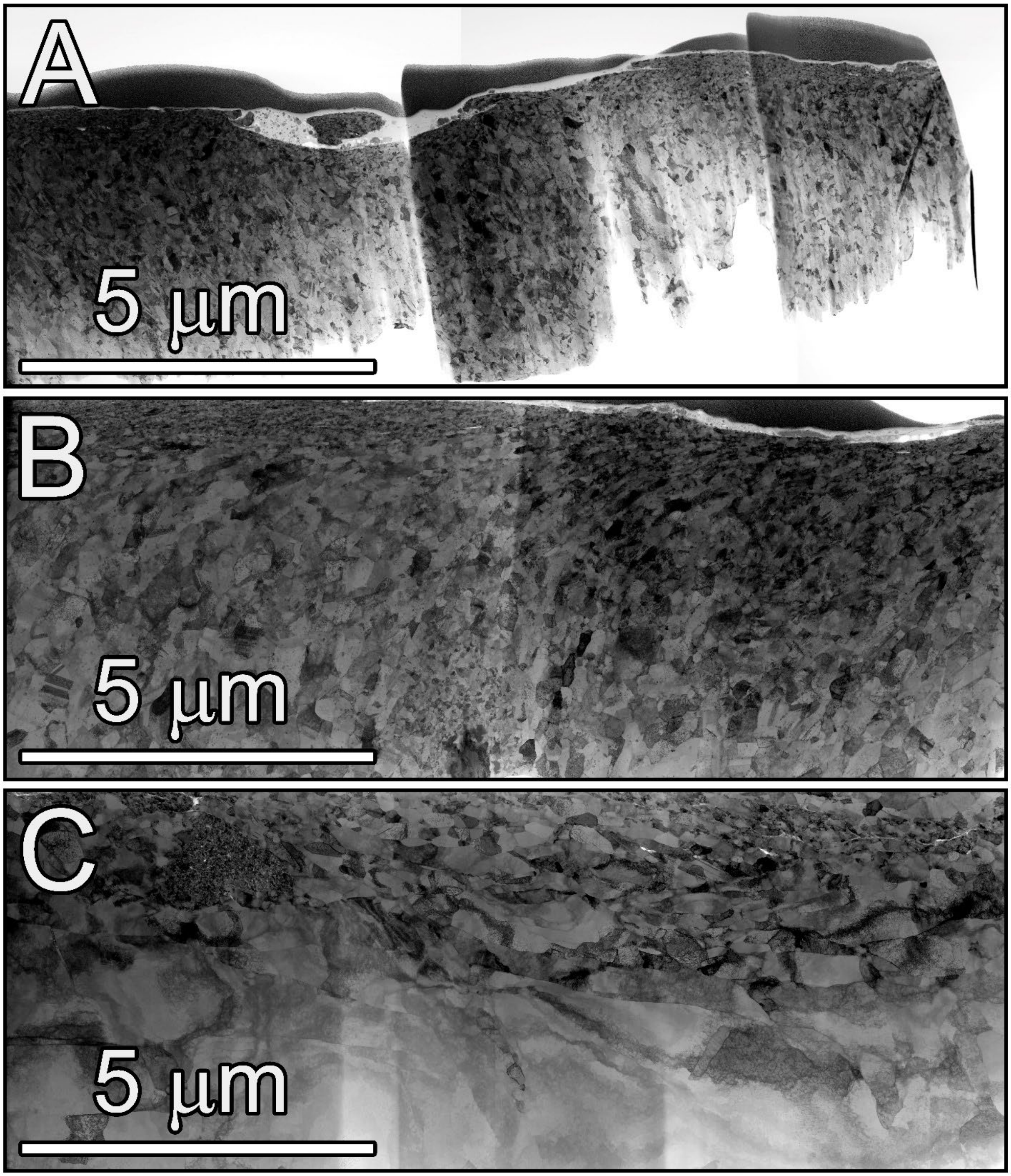

Supplementary Fig. 5: Low magnification (ultra-high-resolution) STEM bright-field images of (A) Cu-3Ta, (B) Cu-1Ta, and (C) Cu after the second shock loading. Each image comprises at least 50 square microns of electron-transparent area.

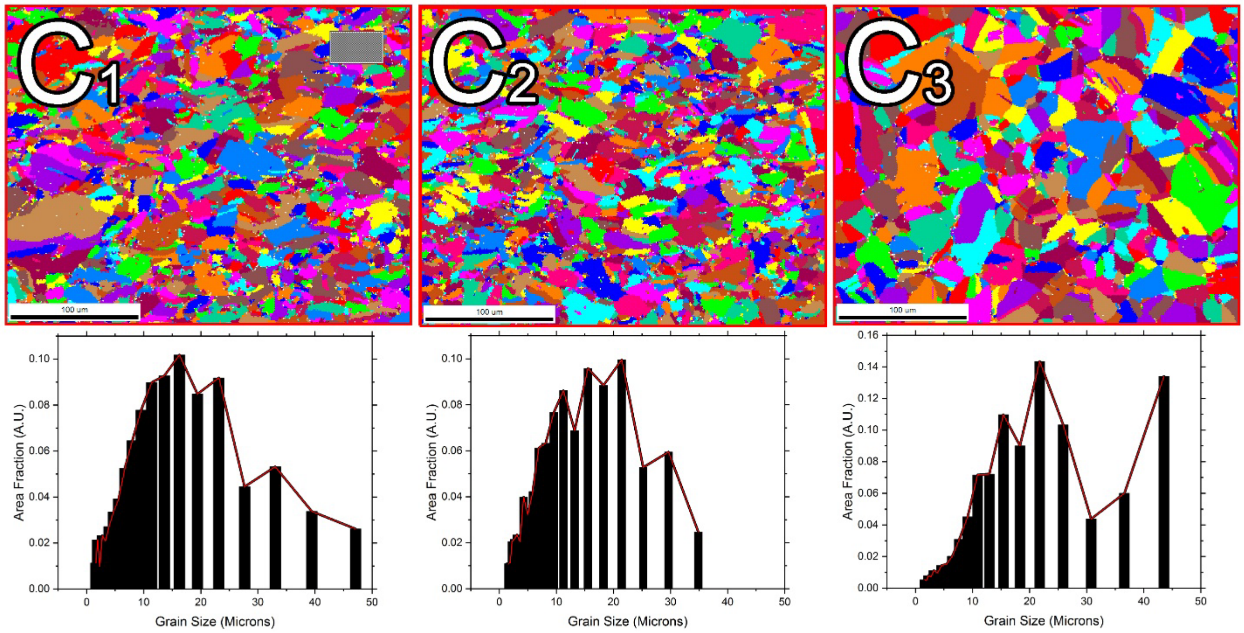

Supplementary Fig. 6: Orientation maps and the corresponding grain size distribution plots for Cu after the second shock loading. (C_1) Impact surface, (C_2) Middle of the sample's through thickness, and (C_3) Rear surface. The gray rectangle in the upper right-hand corner of C_1 represents the size of the six samples shown in Supplementary Fig. 8. Each pixel in the gray box represents the initial grain size in Cu (about 350 nm). The pixels can only be viewed by downloading the full-resolution image available online. Source data are provided as a Source Data file.

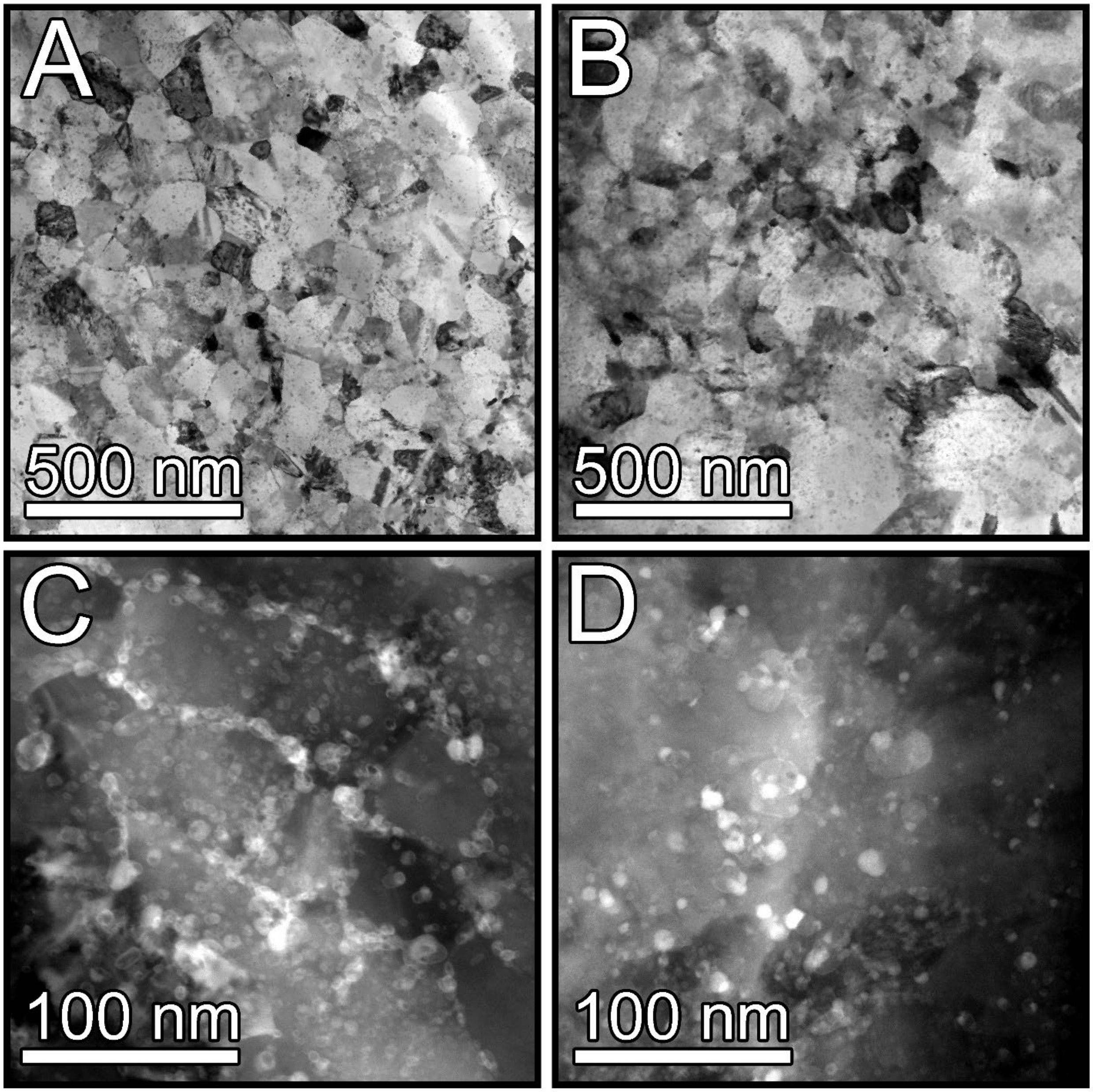

Supplementary Fig. 7: As-received microstructure of Cu-Ta alloys. (A and C) Cu-3Ta and (B and D) Cu-1Ta.

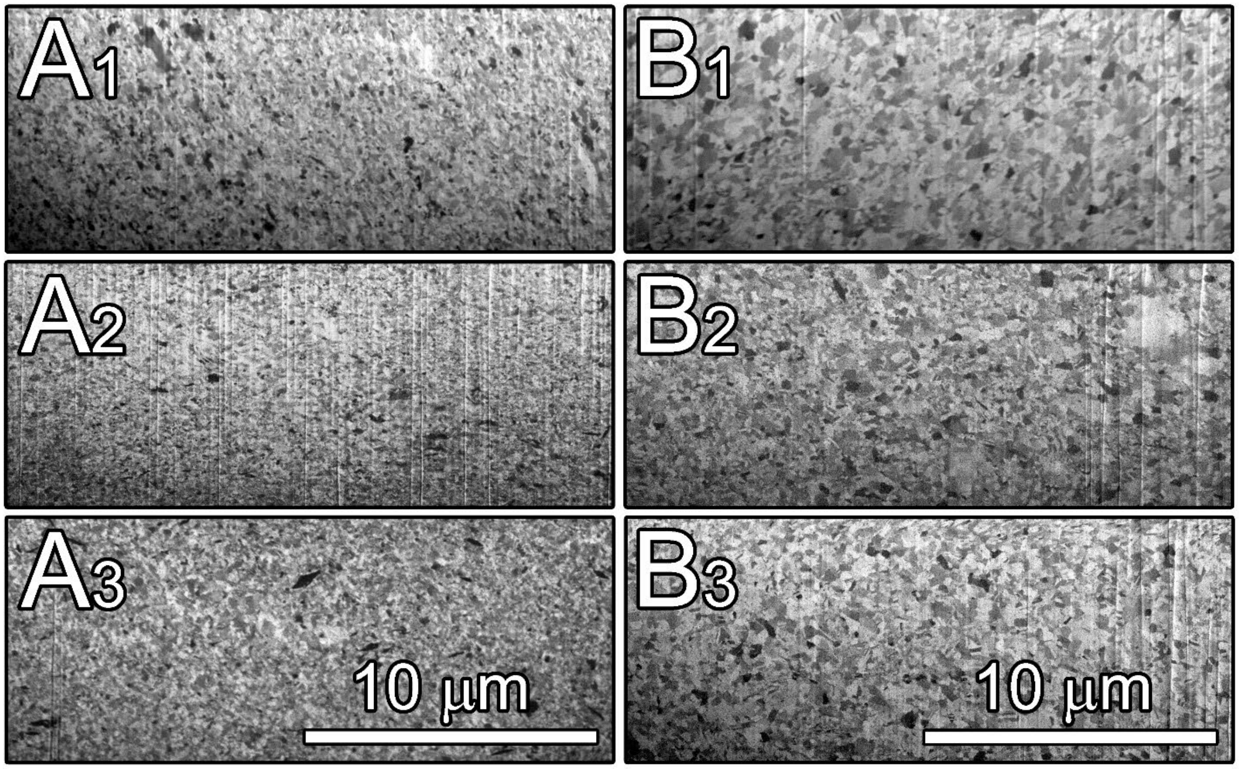

Supplementary Fig. 8: Ion contrast images showing the grain structure in Cu-3Ta and Cu-1Ta, respectively, after a second shock loading at the impact surfaces (A_1 and B_1), at the middle of the through-thickness (A_2 and B_2) and the rear surfaces (A_3 and B_3) of the sample. Note that the grain structure in Cu-3Ta has very few areas with possible abnormal grain growth, while Cu-1Ta has more areas of grain growth.

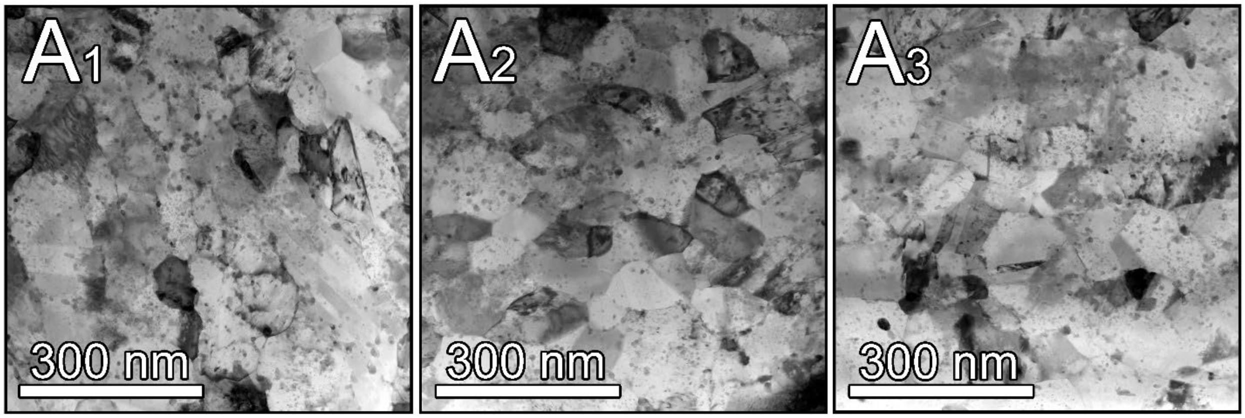

Supplementary Fig. 9: STEM bright-field images showing the microstructure of NC Cu-3Ta after the second shock loading. (A_1) Impact surface, (A_2) Middle of through-thickness, and (A_3) Rear surface of the sample. The microstructures of all three regions exhibit few defects.

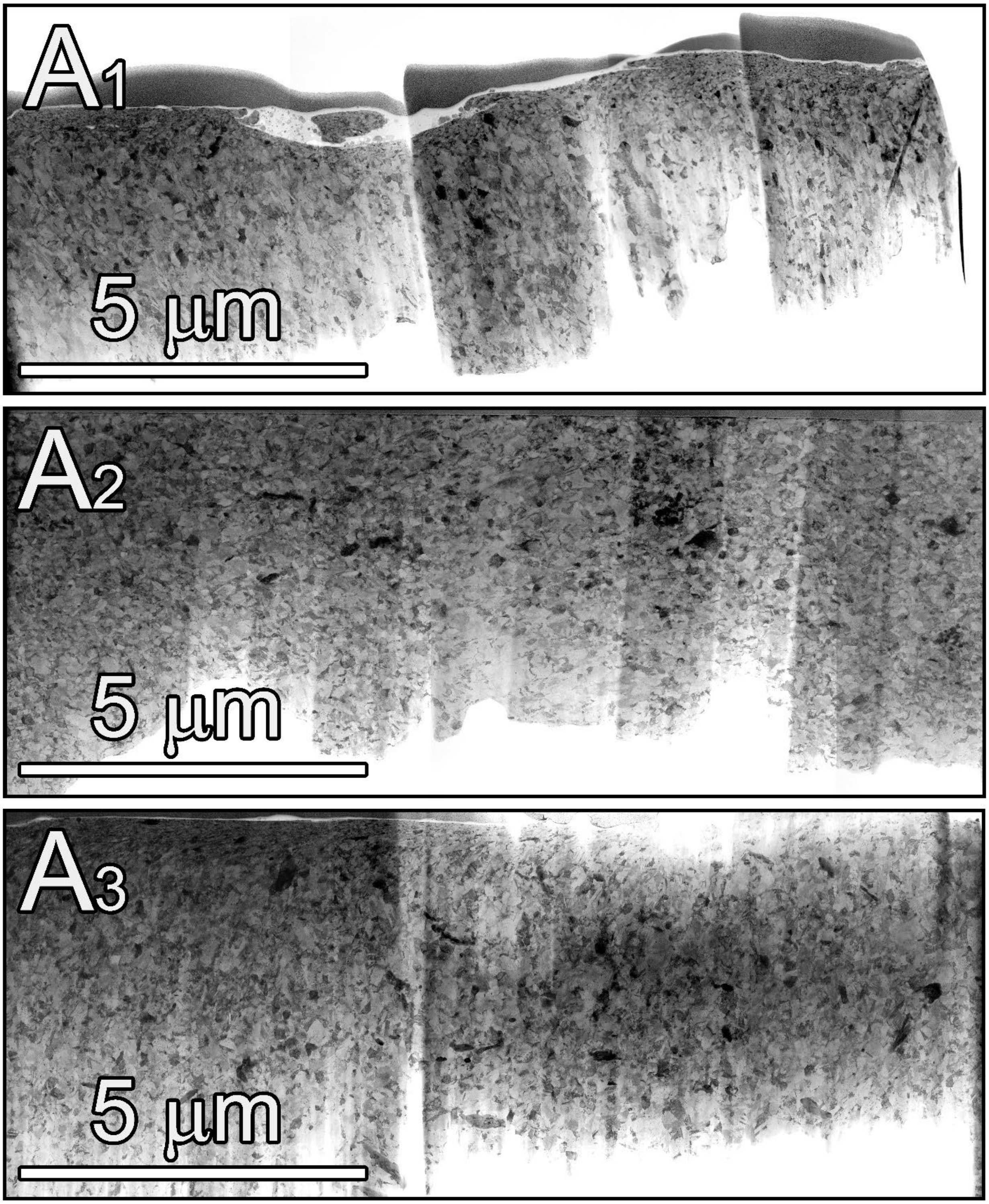

Supplementary Fig. 10: Low magnification ultra-high-resolution STEM bright-field images of Cu-3Ta after the second shock loading at the (A_1) impact surface, (A_2) middle of through-thickness, and (A_3) rear surface of the sample. The microstructures of all three regions have few defects. Each image comprises at least 50 square microns of electron

transparent area. Note the microstructural gradients within the first 0.5 microns of the free surface for the front and back faces of the sample but not in the middle of the film. The gradient effect is caused by intense frictional forces resulting from the interaction of the impact plate with the front face of the sample. These forces also occur between the sample's rear face and the backing plate during shock loading (refer to Supplementary Fig. 14).

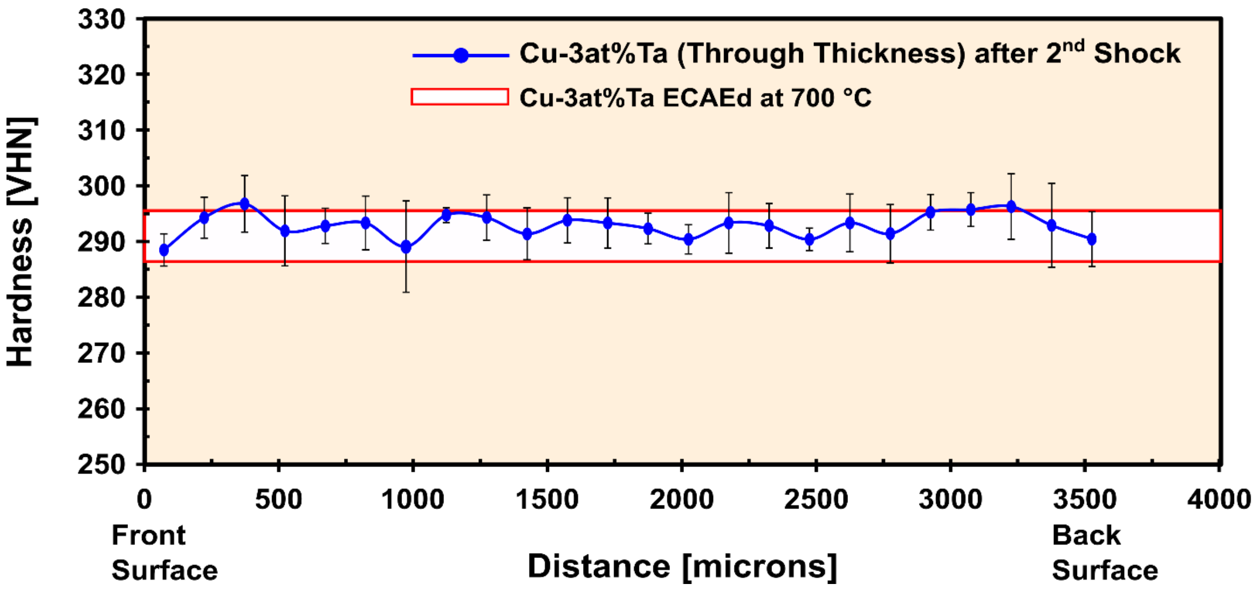

Supplementary Fig. 11: Plot of through thickness (from the front surface to back surface) hardness for Cu-3Ta after 2nd shock loading with the standard deviation for as-ECAE processed Cu-3Ta's hardness overlaid as the white box outlined in red. Source data are provided as a Source Data file.

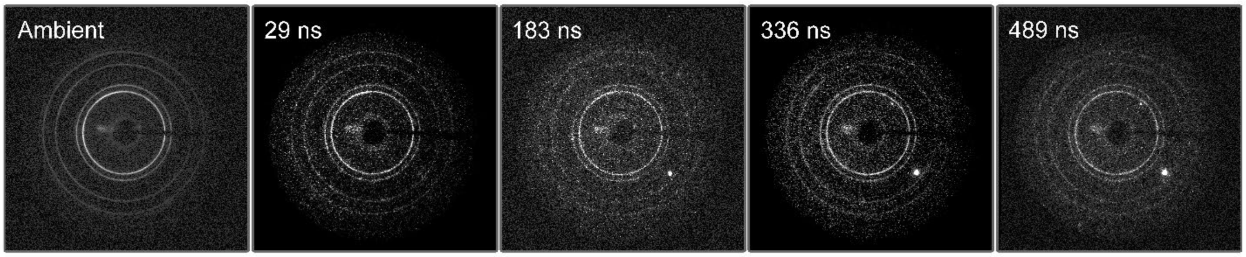

Supplementary Fig. 12: X-ray diffraction for Cu-1Ta during APS experiments from ambient through 489 ns.

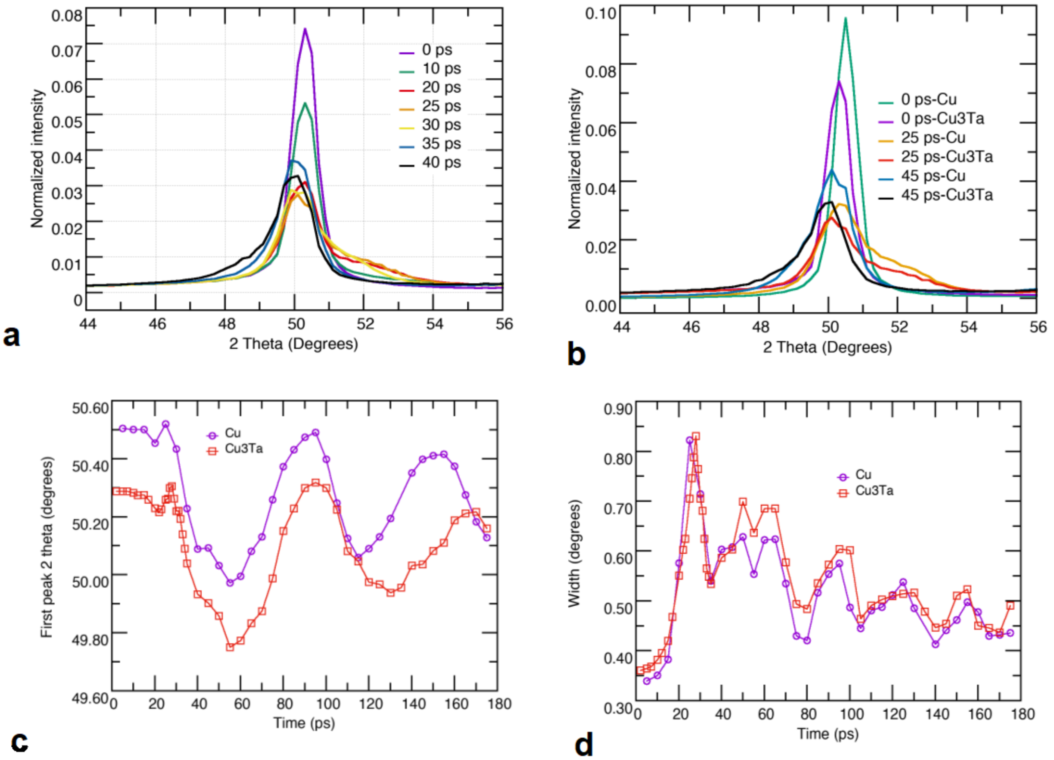

Supplementary Fig. 13: Molecular dynamics simulation of X-ray diffraction during plastic deformation and recovery under shock loading of Cu and the Cu-3Ta alloy. (A) Time evolution of the (111) diffraction peak for Cu-3Ta, showing its broadening and shift to the right followed by narrowing and returning to the pre-shock diffraction angle due to the plastic recovery. (B) Comparison of diffraction peaks for Cu-3Ta and Cu. The diffraction angle of the first peak (C) and the peak width (D) in Cu-3Ta and Cu show variations in time corresponding to the compression, tension, and recovery periods. The diffraction data was collected from the entire simulation sample. Source data are provided as a Source Data file.

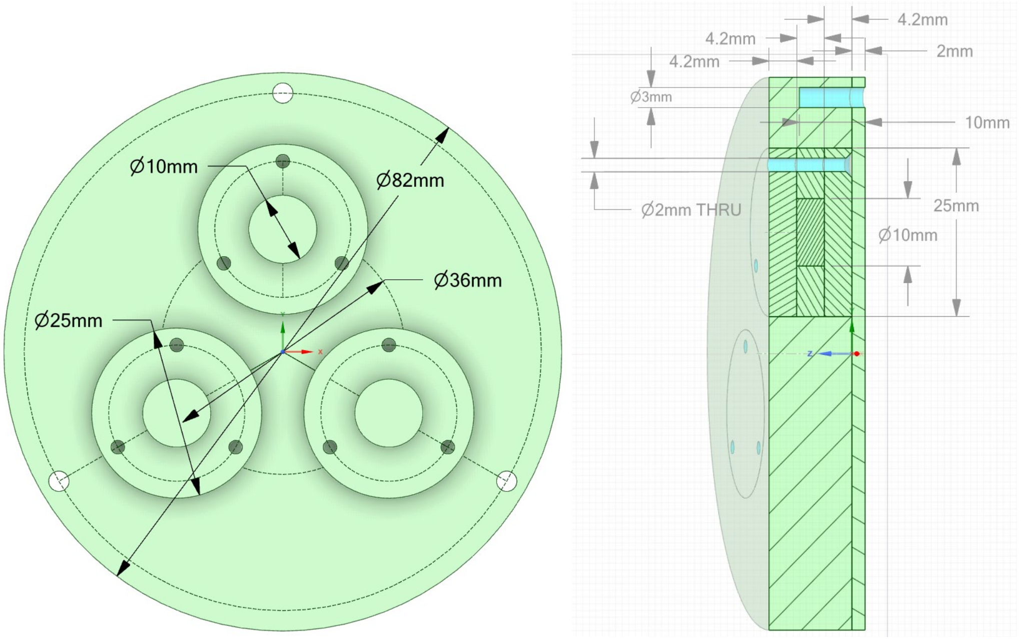

A **B**

Supplementary Fig. 14: (A) Image of the impact face of the three soft recovery capsules. (B) A cross-section of the target with the location of the sample is shown as the 10 mm diameter by 4.2 mm thick central puck in each of the three 25 mm diameter capsules.

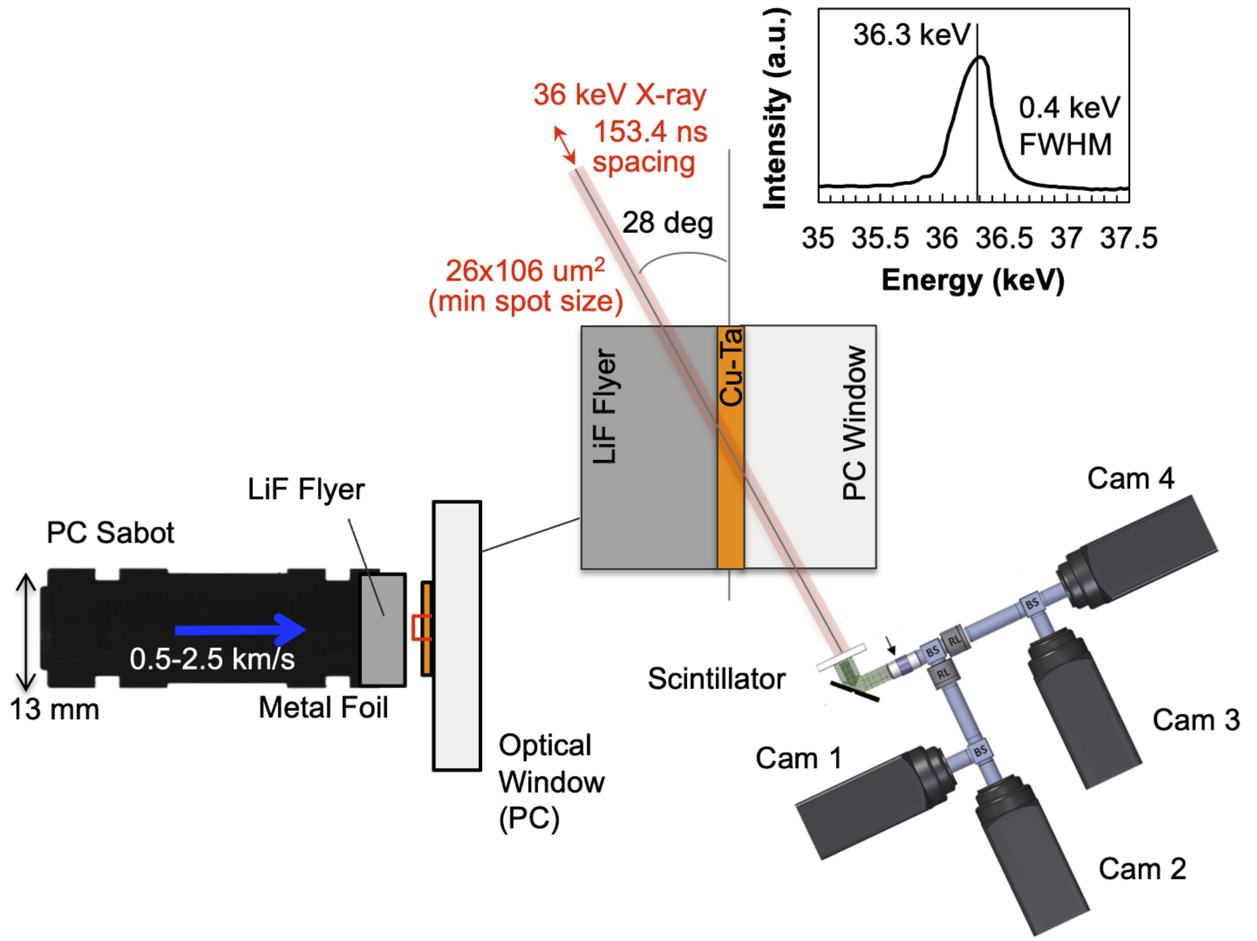

Supplementary Fig. 15: Experimental setup for shock testing and time-resolved X-ray diffraction.
